# Differential regulation of progranulin derived granulin peptides

**DOI:** 10.1186/s13024-021-00513-9

**Published:** 2022-02-04

**Authors:** Tingting Zhang, Huan Du, Mariela Nunez Santos, Xiaochun Wu, Mitchell D. Pagan, Lianne Jillian Trigiani, Nozomi Nishimura, Thomas Reinheckel, Fenghua Hu

**Affiliations:** 1 Department of Molecular Biology and Genetics, Weill Institute for Cell and Molecular Biology, Cornell University, 345 Weill Hall, Ithaca, NY 14853 USA; 2 Nancy E. and Peter C. Meining School of Biomedical Engineering, Cornell University, Ithaca, NY 14853 USA; 3 Institute of Molecular Medicine and Cell Research, Medical Faculty and BIOSS Centre for Biological Signaling Studies, Albert-Ludwigs-University Freiburg, 79104 Freiburg, Germany

**Keywords:** Frontotemporal lobar degeneration (FTLD), Progranulin (PGRN), Granulin, Cathepsin, Glycosylation, Lysosome

## Abstract

**Background:**

Haploinsufficiency of progranulin (PGRN) is a leading cause of frontotemporal lobar degeneration (FTLD). PGRN is comprised of 7.5 granulin repeats and is processed into individual granulin peptides in the lysosome. However, very little is known about the levels and regulations of individual granulin peptides due to the lack of specific antibodies.

**Results:**

Here we report the generation and characterization of antibodies specific to each granulin peptide. We found that the levels of granulins C, E and F are regulated differently  compared to granulins A and B in various tissues. The levels of PGRN and granulin peptides vary in different brain regions and the ratio between granulins and PGRN is highest in the cortical region in the adult male mouse brain. Granulin-A is localized in the lysosome in both neurons and microglia and its levels in microglia increase under pathological conditions. Interestingly,  the levels of granulin A in microglia change correspondingly with PGRN in response to stroke but not demyelination. Furthermore, deficiency of lysosomal proteases and the PGRN binding partner prosaposin leads to alterations in the ratios between individual granulin peptides. Granulins B, C and E are heavily glycosylated and the glycosylation patterns can be regulated under.

**Conclusion:**

Our results support that the levels of individual granulin peptides are differentially regulated under physiological and pathological conditions and provide novel insights into how granulin peptides function in the lysosome.

**Supplementary Information:**

The online version contains supplementary material available at 10.1186/s13024-021-00513-9.

## Background

Progranulin (PGRN) protein, encoded by the granulin (*GRN*) gene, has been implicated in several neurodegenerative diseases [1, 2]. Haplo-insufficiency of the protein, due to heterozygous mutations in the *GRN* gene, is a leading cause of frontotemporal lobar degeneration with TDP-43 aggregates (FTLD-TDP) [3–5]. Homozygous PGRN mutations result in neuronal ceroid lipofuscinosis (NCL), a lysosomal storage disorder [6, 7]. PGRN is known as a secreted glycoprotein of 7.5 granulin repeats [1, 2, 8]. However, accumulating evidence has suggested a critical role of PGRN in the lysosome [8, 9]. PGRN deficiency has been shown to result in lysosome abnormalities with aging [10, 11]. At the molecular and cellular level, PGRN is a lysosome resident protein [12] and *GRN* is transcriptionally co-regulated with many essential lysosomal genes by the transcriptional factor TFEB [13, 14]. PGRN interacts with another lysosomal protein prosaposin (PSAP) to facilitate each other’s lysosomal trafficking [12, 15, 16]. Within the lysosome, PGRN has been shown to get processed to granulin peptides by cathepsins [17–19]. These granulin peptides have been proposed to possess unique biological activities, in a way similar to the saposin peptides derived from PSAP, which function as activators for enzymes involved in glycosphingolipid degradation [20]. In line with this, PGRN and granulin peptides have been shown to regulate the activities of several lysosome enzymes, including cathepsin D [21–24] and glucocerebrosidase [25–27]. Despite these studies, very little is known about how granulin peptides are regulated in the lysosome due to the lack of specific antibodies to each individual peptide.

## Results

### Generation of antibodies to each individual granulin peptide

**Fig. 1:**
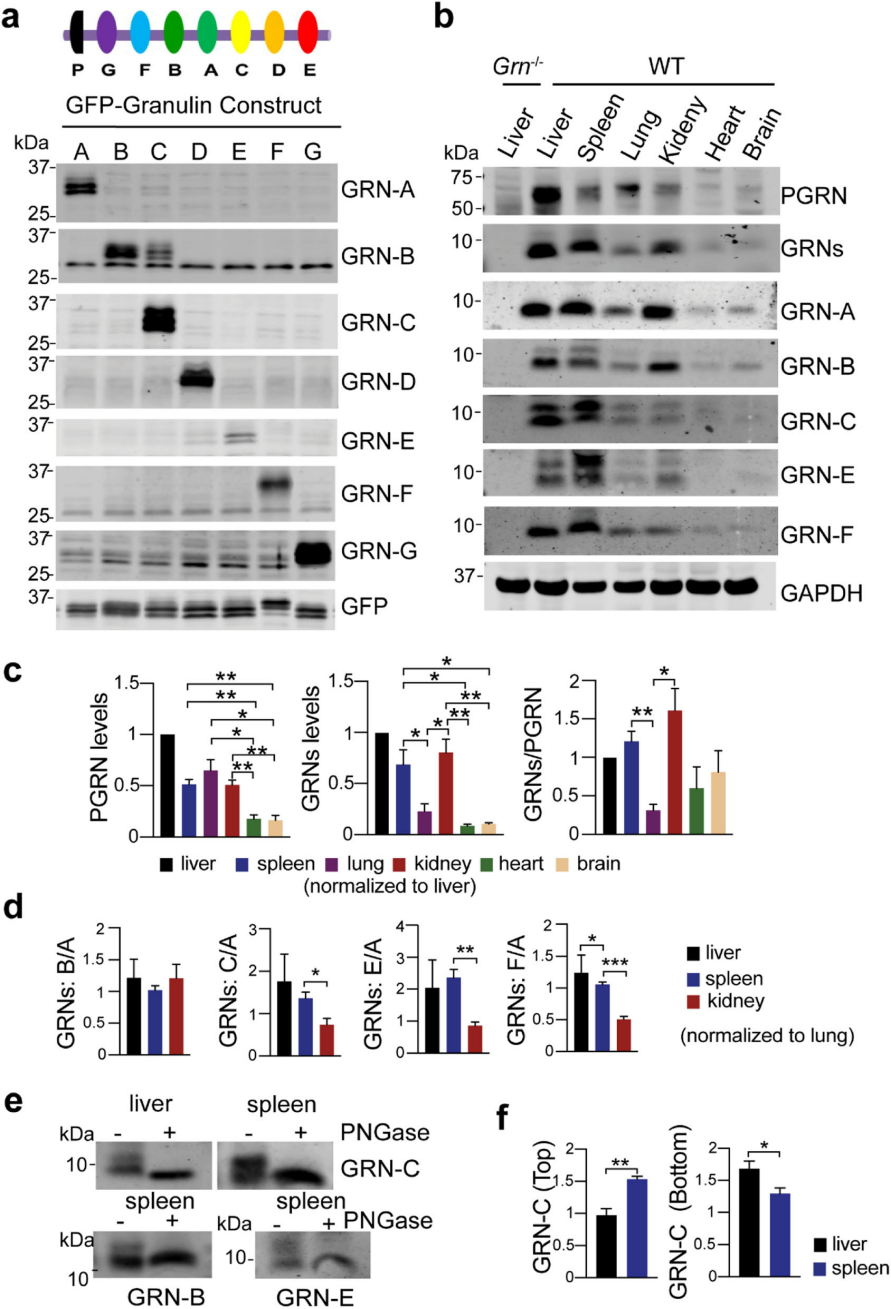
Characterization of granulin antibodies and analysis of granulin levels in tissue lysates. **a** HEK293T lysates containing GFP or GFP tagged mouse granulins were probed with antibodies against each individual granulin peptide as indicated. Diagram of PGRN structure was shown at the top. **b** Western blot analysis of different tissues lysates from 4 to 5 months old WT and *Grn*^*−/−*^ mice with antibodies against each individual granulin, granulin A (GRN-A), granulin B (GRN-B), granulin C (GRN-C), granulin E (GRN-E) and granulin F (GRN- F) as indicated. Full length PGRN and total PGRN-derived granulins (GRNs) were detected by commercial sheep anti-mouse PGRN antibodies (R&D). Mixed male and female mice were used for this analysis. **c** Quantification of experiment in (**b**). The ratio between PGRN and GAPDH; granulins and GAPDH and between granulins and full-length PGRN was quantified and normalized to that in the liver sample on the same gel (set as 1). Data presented as mean ± SEM. *n* = 3. *, *p* < 0.05, **, *p* < 0.01, ***, *p* < 0.001, ****, *p* < 0.0001, unpaired two-tailed Student’s *t*-test. **d** Quantification of the ratios between granulins B/C/E/F and granulin A. The band intensities of different granulins were measured and normalized to GAPDH. The ratios between granulins B/C/E/F and granulin A were calculated and normalized to the lung sample on the same gel (set as 1). Data presented as mean ± SEM. The ratio of GRN-C (*p* = 0.04), GRN-E (*p* = 0.0057), GRN-F (*p* = 0.0006), but not GRN-B (*p* = 0.4586) versus GRN-A is significantly lower in kidney than that in lung. *, *p* < 0.05, **, *p* < 0.01, ***, *p* < 0.001, unpaired two-tailed Student’s *t*-test. **e** Granulin B, C, and E are glycosylated. Spleen or liver lysates from WT mice were immunoprecipitated using anti-granulin B, C, or E antibodies and the immunoprecipitates were treated with PNGase F. **f** Quantification of two differentially glycosylated forms of granulin C for experiments in (**b**). The ratio between top or bottom bands of granulin C and GAPDH in liver and spleen lysates was quantified and normalized to their levels in the lung. Data presented as mean ± SEM. *n* = 4. Top bands (*p* = 0.0023), bottom bands (*p* = 0.04), *, *p* < 0.05, **, *p* < 0.01, unpaired two-tailed Student’s *t*-test

To generate antibodies specific to each granulin peptide, we purified recombinant GST tagged mouse granulin peptides from bacteria (Table S1). These proteins were then used to immunize rabbits to generate polyclonal antibodies. To test the specificity of these antibodies toward each individual granulin peptide, we expressed individual mouse granulin peptides in HEK293T cells with an N-terminal signal sequence followed by a GFP tag. HEK293T lysates containing GFP tagged granulins were then used in western blot analysis to determine the specificity of the antibodies against each individual granulin peptide. All the granulin antibodies specifically recognized their respective granulin peptide, except the granulin B antibody, which exhibits weak cross-reactivities with granulin C (Fig. 1a).

Next, we determined whether these antibodies could detect endogenous granulin peptide using liver lysates from adult WT and *Grn*^*−/−*^ mice. Specific signals around 10 kDa were successfully detected in the WT lysates but not in the *Grn*^*−/−*^ samples with granulin A, B, C, E, and F antibodies (Fig. 1b). Unfortunately, granulin D and G antibodies cannot detect endogenous granulin peptides, although they recognize overexpressed granulin peptides efficiently. Thus, we focused our effort on granulins A, B, C, E, and F for the current study.

### Variation in the levels of granulin peptides in different tissues

To determine whether the levels of granulin peptides vary from each other, first, we analyzed the levels of each granulin peptide in different tissues using western blots (Fig. 1b, c). We found that PGRN is highly expressed in the liver, spleen, lung and kidney (Fig. 1b, c). Using the commercial PGRN antibody which preferentially recognizes granulins B, C and F (Fig. S1), a corresponding enrichment of granulins is detected in the liver, spleen and kidney, but not in the lung (Fig. 1b, c). Using antibodies against individual granulins, relatively high levels of granulins A and B in the liver, spleen and kidney but not in the lung were also observed (Fig. 1b, Fig. S2), indicating that PGRN processing or the stability of granulins A and B are different in the lung versus spleen and kidney. Interestingly, while the levels of granulins C, E, and F are also high in the liver and spleen and low in the lung, their levels are relatively low in the kidney, as shown by a significant decrease in the ratio between granulins C/E/F and granulin A in the kidney compared to that in the liver and spleen (Fig. 1b and d). This suggests that the levels of granulin peptides could differ from each other although they are derived from the same precursor. This could be due to differential processing or differences in their stability within the lysosome.

### Glycosylation of granulins B, C, and E

PGRN is predicted to contain 5 N-glycosylation sites with granulin B, C and E each harboring one glycosylation site. Additionally, glycosylation sites in granulins C and E have been mapped by mass spectrometry analysis [28]. Glycosylation is known to play an important role in protein folding and stability as well as protein-protein interaction and signal transduction [29]. In our western blot analysis, two distinct bands have been observed for granulin B, C and E at endogenous levels (Fig. 1b). We speculated that these two bands observed for granulins B, C and E could be peptides with different degrees of glycosylation. To test this, we immunoprecipitated granulin B, C and E peptides with their corresponding antibodies and treated the immunoprecipitates with PNGase F to remove N-glycans. The two bands collapsed to a single band with the lower molecular weight with PNGase F treatment, confirming that granulin B, C and E have two different glycosylated forms (Fig. 1e). More interestingly, the pattern of glycosylation for granulin B and C differs in the spleen lysates versus liver lysates (Fig. 1b,f). In both cases, an increased level of the highly glycosylated form was observed in the spleen compared to the liver, especially for granulin C (Fig. 1b, f).

### Variations in the levels of PGRN and granulin peptides in different brain regions

**Fig. 2:**
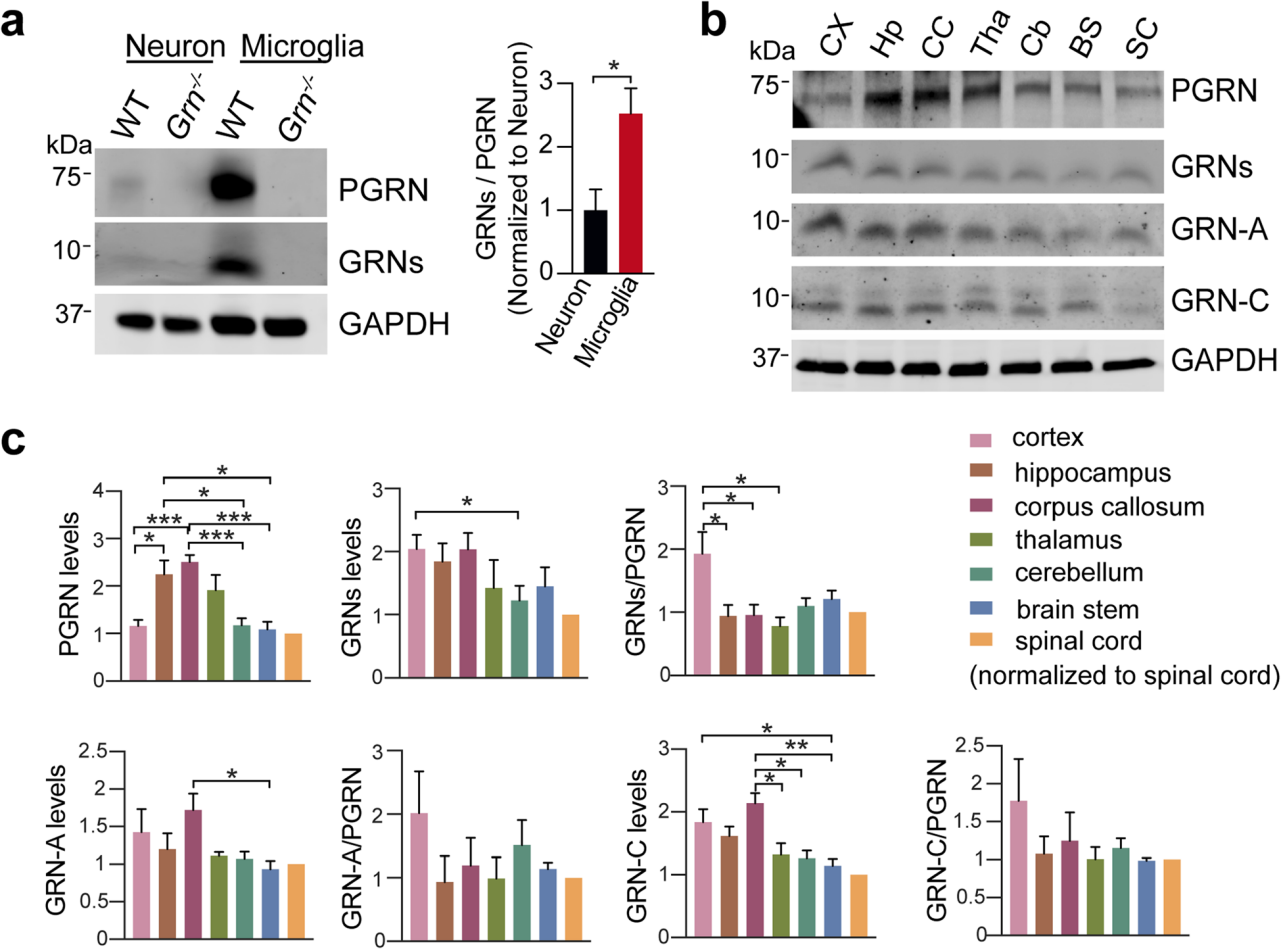
Analysis of PGRN and granulin levels in brain regions and spinal cord. **a** Western blot analysis of primary cortical neuron (DIV14) and microglia lysates using sheep anti-PGRN antibodies. *n* = 4. Data presented as mean ± SEM. *, *p* < 0.05, unpaired two-tailed Student’s *t*-test. **b** Western blot analysis of tissue lysates from 4.5 to 5 months old male WT mice with antibodies against full-length of PGRN and individual granulin A and C as indicated. CX = cortex, Hp = hippocampus, CC = corpus callosum, Tha = thalamus, Cb = cerebellum, BS = brain stem, SC = spinal cord. **c** Quantification of experiment in (**b**). The ratio of each granulin peptide to full-length PGRN was quantified and normalized to the value of spinal cord samples on the same gel (set as 1). *n* = 3–5. Data presented as mean ± SEM. *, *p* < 0.05, **, *p* < 0.01, ***, *p* < 0.001, unpaired two-tailed Student’s *t*-test

Since PGRN is expressed by both neurons and microglia, we wonder if there is a difference in PGRN processing in neuron and microglia. We found that the levels of both PGRN and granulin peptides are much higher in microglia compared to neurons and the ratio of total GRNs to full-length PGRN is 2–3 fold higher in microglia (Fig. 2a).

PGRN is broadly distributed in different brain regions and the spinal cord [30, 31]. Thus, it will be interesting to examine the distribution of granulin peptides in different brain regions. Since granulins A and C show differential regulation and our antibodies against granulins A and C can reliably detect these peptides in the brain lysates, we compared the levels of PGRN and granulins A and C in different regions of male and female mice using western blot analysis (Fig. 2b). In male mice, the levels of full-length PGRN are highest in the hippocampus, corpus callosum, and thalamus, as compared to other regions (Fig. 2b and c). The levels of granulins detected with commercial anti-PGRN antibodies (Fig. S1) and the levels of granulin A and C are also high in the hippocampus and corpus callosum lysates, but relatively lower in the thalamus. In addition, although the levels of full-length PGRN are low in the cortex, the levels of granulins A and C are highest in the cortex (Fig. 2a and b). These results indicate that the levels of both PGRN and granulin peptides vary in different brain regions. This could be caused by differences in microglial density or differences in PGRN expression and processing/stability of granulin peptides in different types of neurons. However, we were unable to obtain consistent results from female mice. In females, both the levels of full-length PGRN and the ratio between individual granulins and PGRN show a high degree of variability from mouse to mouse (Fig. S3). The variability in females could be caused by changes in estrogen levels during the estrous cycle, since PGRN gene expression is known to be regulated by estrogen [30–33]. The levels of PGRN and granulins as well as the GRN/PGRN ratio can be examined at the different stages of the estrous cycle in mice to test the effect of fluctuations in estrogen and other hormones on PGRN levels and processing.

### Granulin A is localized to the lysosome in both neurons and microglia

**Fig. 3:**
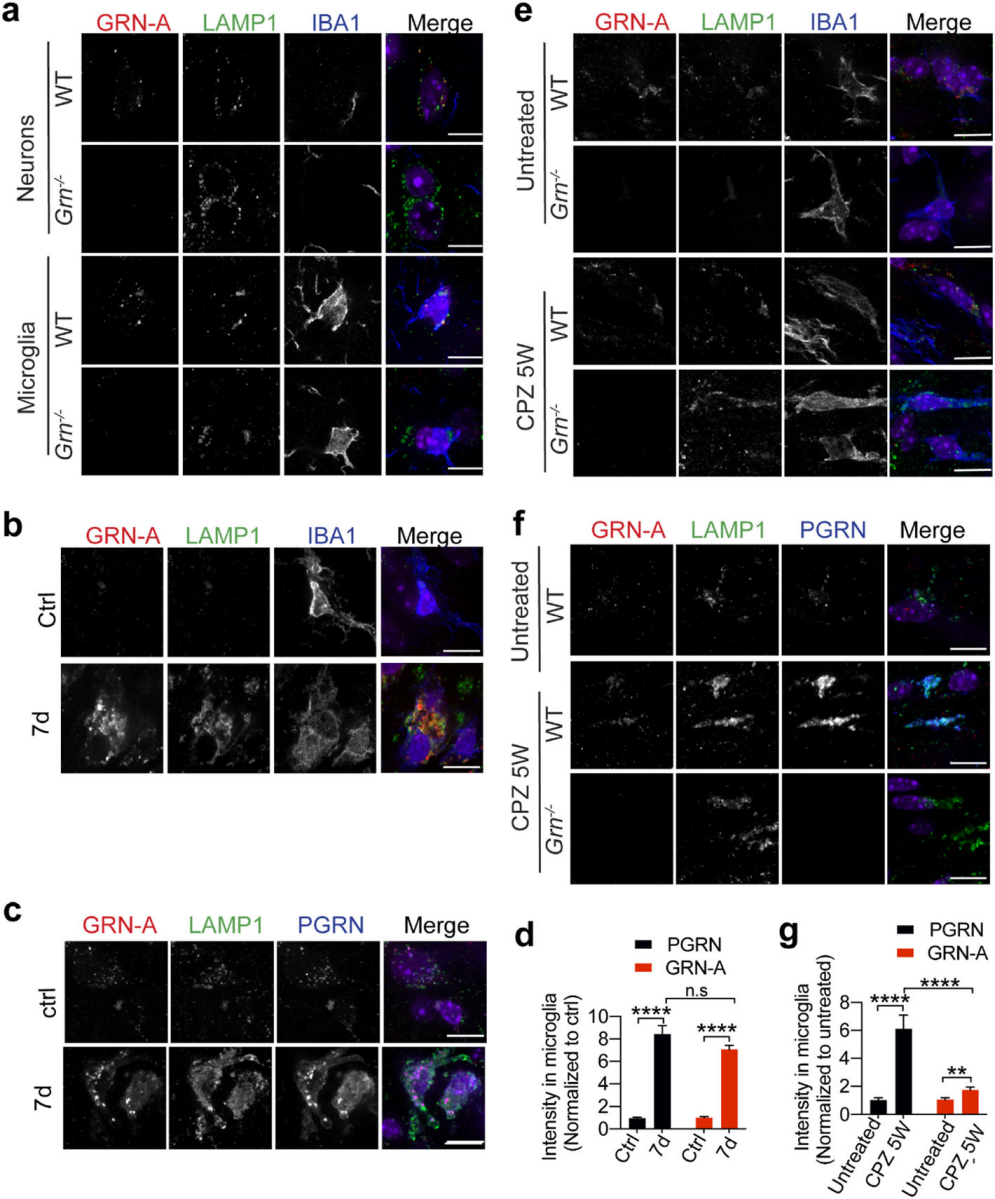
Granulin A is localized in the lysosome in neurons and microglia. **a** Immunostaining of GRN-A, LAMP1 and IBA1 in brain sections from 4 months old WT and *Grn*^−/−^ mice. Representative images from the cortex were shown. Scale bar = 10um. **b, c, d** Immunostaining of GRN-A, LAMP1 and IBA1 or GRN-A, LAMP1and PGRN in brain sections from 4 months old WT mice untreated or treated with Rose Bengal and laser illumination to induce stroke (7 days after treatment). Representative images in the lesion site were shown. Scale bar = 10um. PGRN and GRN-A signals in IBA1 positive microglia were quantified by Image J. 40–50 individual microglia were quantified from multiple brain sections from 2 independent mice for each condition. Data presented as mean ± SEM. ****, *p* < 0.0001, n.s, not significant, unpaired two-tailed Student’s *t*-test. **e, f, g** Immunostaining of GRN-A, LAMP1 and IBA1 or GRN-A, LAMP1and PGRN in brain sections from 15-week old WT and *Grn*^−/−^ mice untreated or treated with cuprizone for 5 weeks (CPZ 5 W). Representative images from the corpus callosum region were shown. Scale bar = 10um. PGRN and GRN-A signals in IBA1 positive microglia were quantified by Image J. 40–50 individual microglia were quantified from multiple brain sections from 2 independent mice for each condition. Data presented as mean ± SEM. **,*p* < 0.01, ****, *p* < 0.0001, unpaired two-tailed Student’s *t*-test

In addition to western blot analysis, it will be important to examine the levels and localization of individual granulins in different cell types using immuno-staining. To this end, we have optimized conditions to detect mouse granulin A in mouse brain sections by pre-incubating the antibodies with brain sections from PGRN deficient mice to get rid of non-specific signals. We have detected specific signals for granulin-A in the lysosome in both neurons and microglia, as shown by colocalization with LAMP1 (Fig. 3a). Since PGRN is known to get upregulated in microglia in many pathological conditions [8], we examined granulin-A levels in microglia upon stroke and cuprizone-induced demyelination. Increased levels of granulin A were detected in microglia after stroke (Fig. 3b, d), and granulin-A signals overlapped with signals detected by the commercial anti-PGRN antibodies and anti-LAMP1 antibodies (Fig. 3c), indicating increased levels of lysosomal granulin-A upon microglial activation in response to stroke. Interestingly, only a very modest increase of GRN-A levels was detected in microglia in the corpus callosum region in response to demyelination, although there is a significant increase in PGRN levels in microglia (Fig. 3e-g). These indicated that PGRN processing or stability of granulin A might be regulated differently in microglia with different activation states.

### Regulation of PGRN processing and granulin levels by lysosomal proteases

**Fig. 4:**
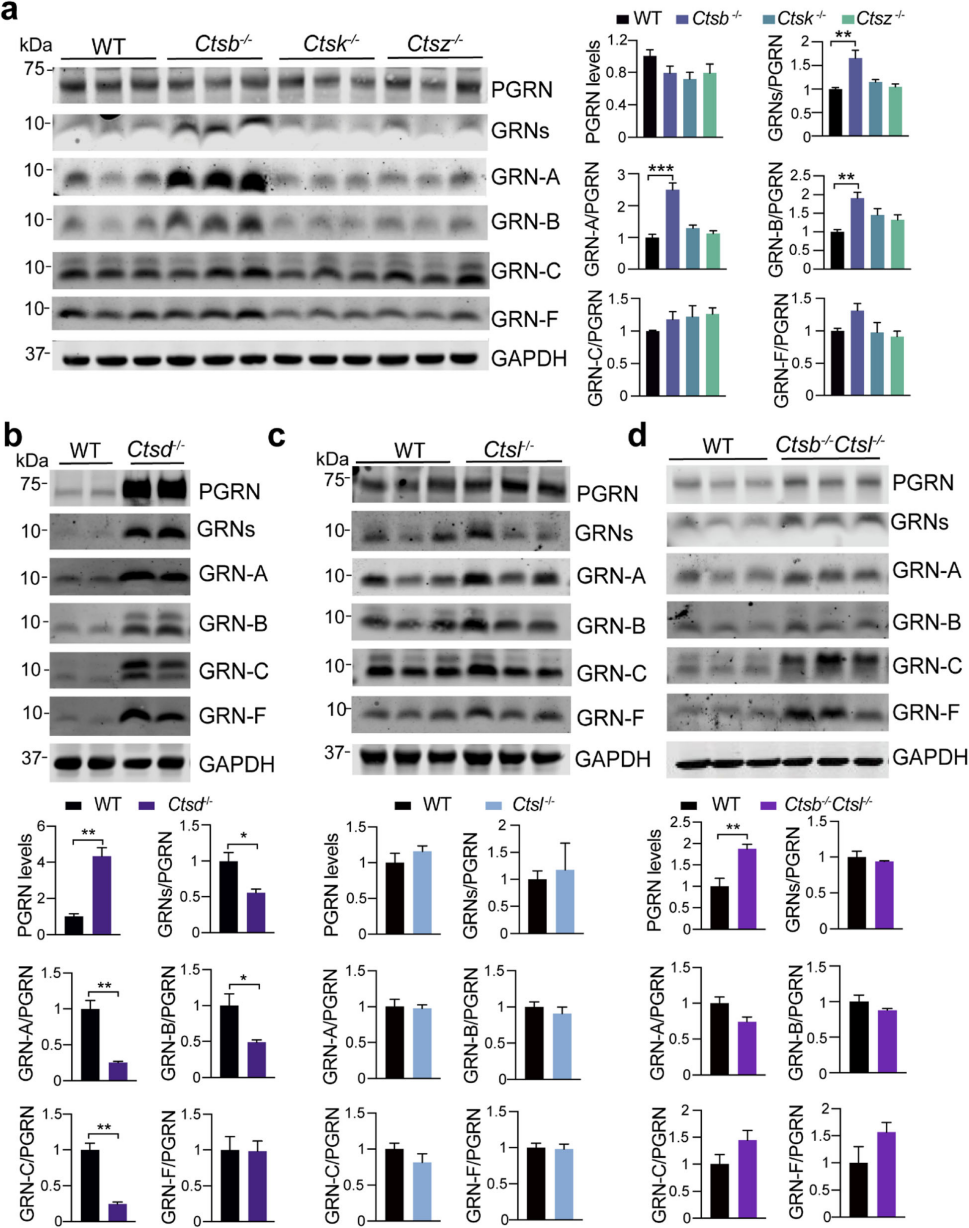
Analysis of PGRN and granulin levels in brain lysates from cathepsin deficient mice. **a** Western blot analysis of cortical lysates from 5 months old WT, *Ctsb*^*−/−*^, *Ctsk*^−/−^ and *Ctsz*^−/−^ mice with antibodies against each granulin peptide as indicated. The ratio between each granulin peptide to full-length PGRN was quantified and normalized to WT. Data presented as mean ± SEM. *n* = 3. Significant increased levels of GRNs (*p* = 0.0032, one-way ANOVA with Bonferroni’s post hoc comparisons), GRN-A (*p* = 0.0002), GRN-B (*p* = 0.0089) in *Ctsb−/−* cortical lysates compared to WT. *, *p* < 0.05, **, *p* < 0.01, ***, *p* < 0.001. Mixed male and female mice were used for this analysis. **b-d** Western blot analysis of cortical lysates from 2 weeks old *Ctsd*^−/−^ mice (**b**), 5 months old *Ctsl*^−/−^ mice (**c**), 3 weeks old *Ctsb*^*−/−*^*Ctsl*^−/−^ mice (**d**), and age-matched WT mice using antibodies against each granulin peptide as indicated. The ratio between PGRN and GAPDH as well as between each granulin peptide and full-length PGRN was quantified and normalized to WT. Data presented as mean ± SEM. n = 3–5. The levels of PGRN are increased in *Ctsd*−/− (*p* = 0.0025) and *Ctsb*−/−*l*−/− (*p* = 0.0071) cortical lysates compared to WT. The ratio of total GRNs (*p* = 0.0286), GRN-A (*p* = 0.0033), GRN-B (*p* = 0.0373), GRN-C (*p* = 0.0015), but not GRN-F (*p* = 0.9453), versus full length of PGRN is decreased in the *Ctsd*^*−/−*^ lysates. Data were analyzed by unpaired two-tailed Student’s *t*-test. *, *p* < 0.05, **, *p* < 0.01. Mixed male and female mice were used for this analysis

Lysosomal proteases, such as cathepsins, have been shown to play a role in PGRN processing. The cysteine protease, cathepsin L, was shown to cleave PGRN to granulin peptides efficiently in vitro [17–19]. However, how lysosomal proteases regulate PGRN cleavage in vivo remains unclear. Using the granulin-specific antibodies, we analyzed the levels of each granulin peptide in the cortex of mice deficient in individual lysosomal proteases, including cathepsin B, D, L, K, and Z (Fig. 4a, b, c, Table S2). Ablation of most of these cathepsins individually does not seem to have a significant effect on the levels of full-length PGRN and total granulins, except cathepsin B and cathepsin D. The levels of both PGRN and granulin peptides are significantly upregulated in *Ctsd*^*−/−*^ cortical lysates (Fig. 3b), partly due to transcriptional up-regulation as reported previously [32]. The ratio of granulins A, B, C, but not granulin F, to full-length PGRN is decreased in the *Ctsd*^*−/−*^ lysates, suggesting that ablation of cathepsin D affects PGRN processing or the stability of a subset of granulins (Fig. 4b). Interestingly, a significant increase in the levels of the heavily glycosylated form of granulin C was observed in *Ctsd*^*−/−*^ lysates (Fig. 4b), suggesting possible dysfunction of lysosomal glycosidases in response to cathepsin D loss. Although cathepsin B deficiency does not have any obvious effect on the levels of PGRN, it leads to a significant increase in the levels of granulin A and B without any obvious effects on granulin C and F (Fig. 4a), suggesting that cathepsin B might specifically regulate the generation or stability of granulin A and B in the lysosome. Despite results from in vitro studies supporting an important role of cathepsin L in PGRN processing [17–19], ablation of cathepsin L does not have any obvious effect on the levels of PGRN and granulin peptides in the brain (Fig. 4c). Cathepsins B and L are known to have overlapping functions. In cortical lysates from cathepsin B and L double knockout (*Ctsb*^*−/−*^ *Ctsl*^*−/−*^) mice, levels of full-length PGRN are significantly increased (Fig. 4d), possibly due to transcriptional upregulation caused by severe lysosomal abnormalities in these mice [33, 34]. However, the ratio between individual granulin peptides to full-length PGRN is not altered in *Ctsb*^*−/−*^ *Ctsl*^*−/−*^ brain lysates, indicating that none of these two cysteine proteases is essential for PGRN processing in vivo. It should be noted that the levels of other lysosomal proteases are likely to be changed upon the loss of one or more proteases [35]. Thus, other proteases may get upregulated to process PGRN in the absence of cathepsins B and L. Heavily glycosylated form of granulin C accumulates in *Ctsb*^*−/−*^ *Ctsl*^*−/−*^ cortical lysates similar to that in *Ctsd*^*−/−*^ mice, indicating changes in the activities of lysosomal glycosidases upon lysosomal dysfunction. Interestingly, the glycosylation pattern of granulin B does not appear to be affected in *Ctsb*^*−/−*^ *Ctsl*^*−/−*^ cortical lysates, suggesting the glycosylation of granulin B and C are subject to different regulations.

### Regulation of PGRN processing and granulin levels by prosaposin (PSAP)

**Fig. 5:**
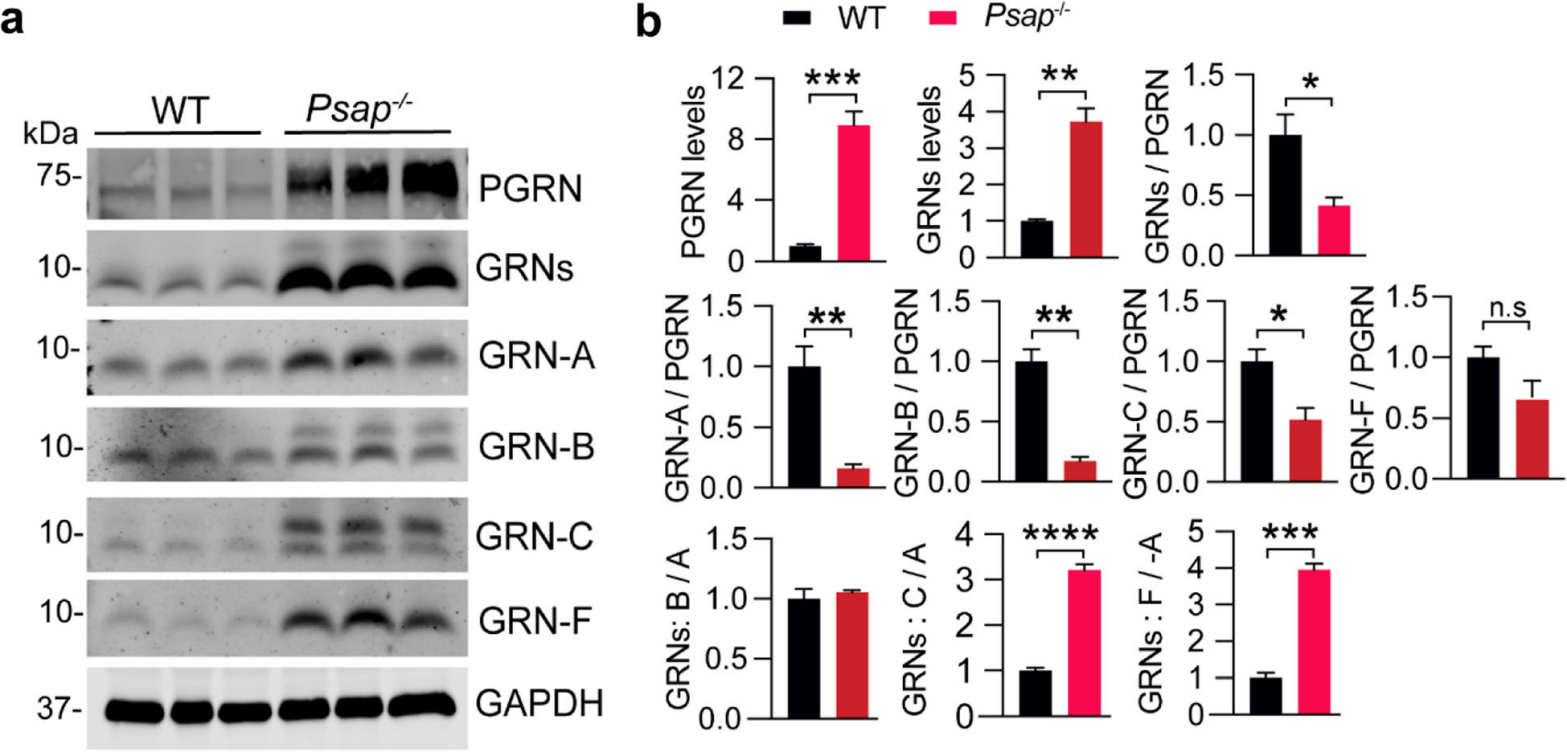
Analysis of PGRN and granulin levels in brain lysates from PSAP deficient mice. **a, b** Western blot analysis of cortical lysates from 3-week old WT and *Psap*^−/−^ mice with antibodies against each granulin peptide and full-length PGRN as indicated. The ratio between PGRN or total granulins or each granulin peptide and GAPDH as well as between granulins and full-length PGRN was quantified and normalized to WT. The ratios between granulins B/C/E/F and granulin A were quantified and shown in (**b**). Data presented as mean ± SEM. *n* = 3. The levels of PGRN (*p* = 0.0009) and granulins (*p* = 0.0017) are increased in *Psap*^−/−^ mouse cortical lysates compared to WT. The ratio of total GRNs (*p* = 0.0339), GRN-A (*p* = 0.0078), GRN-B (*p* = 0.0016), GRN-C (*p* = 0.025), but not GRN-F (*p* = 0.093), versus full length of PGRN is decreased in the *Psap*^*/−*^ lysates. Data were analyzed by unpaired two-tailed Student’s *t*-test. *, *p* < 0.05, **, *p* < 0.01, ***, *p* < 0.001. Mixed male and female mice were used for this analysis

PGRN interacts with another lysosomal protein prosaposin (PSAP) to facilitate each other’s lysosomal trafficking [12, 15, 16]. Within the lysosome, PGRN and PSAP get processed to individual granulins [17–19] or saposins [20], respectively. However, it remains unknown whether the interaction between PGRN and PSAP influences each other’s processing. To determine the role of PSAP on PGRN processing, we examined the levels of PGRN and granulin peptides in cortical lysates from *Psap*^*−/−*^ mice. Due to early lethality and lysosomal abnormalities in *Psap*^*−/−*^ mice [20], there is a significant increase in the levels of both PGRN and granulin peptides because of transcriptional responses. However, the ratio of granulin peptides to full-length PGRN is significantly decreased (Fig. 5a, b), consistent with the fact that PSAP is required for efficient PGRN lysosomal delivery [12, 15, 16]. Interestingly, the fold of increase in granulin C and F levels are much more than that in granulins A and B levels in *Psap*^*−/−*^ mice (Fig. 5b). While granulin F levels are increased proportionally to full length PGRN and the levels of granulin C are also drastically increased, there are very little changes in the levels of granulin A and B, which is reflected in the increased ratio of granulin C to A and of granulin F to A (Fig. 5b). These results further support that the levels of individual granulin peptides can be differentially regulated. In addition, there is a significant increase in the levels of hyper-glycosylated forms of both granulin B and C in the cortical lysates from *Psap*^*−/−*^ mice (Fig. 5a), supporting that glycosylation of granulin B and C are subject to regulation in pathological conditions.

## Discussion

PGRN shares many similarities with its binding partner and travel companion prosaposin (PSAP). By forming a complex, these two proteins facilitate each other’s lysosomal trafficking [12, 15, 16]. In addition, when reaching the lysosome, PGRN and PSAP are processed into granulins and saposins, respectively, through the action of lysosomal proteases [9]. Saposins are key regulators of enzymes involved in the glycosphingolipid degradation pathway [20]. Interestingly, although derived from the same precursor, individual saposins are known to be regulated differently in the lysosome. For example, saposin A and D are the main protein components of lipofuscin found in many lysosomal storage diseases [36], indicating that saposin A and D have distinct biochemical properties in the lysosome compared to saposin B and C. However, not much is known about the function and regulation of granulin peptides in the lysosome. Since granulin peptides are likely to be the functional units of PGRN within the lysosome and PGRN haploinsufficiency in FTLD is known to cause haploinsufficiency of granulin peptides [17], it is critical for us to understand how the levels of individual granulins are regulated. In this manuscript, we report the generation and characterization of antibodies towards each mouse granulin. With these unique antibodies, we have shown that [1] The levels of granulins A and B are differently regulated compared to the levels of granulins C, E and F [2]; Granulins B, C and E are heavily glycosylated and the glycosylation pattern is subject to regulation. Due to the redundancy and cross-regulation of lysosomal proteases, it is challenging to dissect the precise mechanisms involved in PGRN processing. Nevertheless, our results show that cathepsin B might play a role in regulating the levels of granulins A and B (Fig. 4a) and ablation of cathepsin D leads to decreased ratios of granulins A, B and C to PGRN (Fig. 4b) (Table S2). Ablation of PSAP results in a general reduction of PGRN processing, due to lysosomal trafficking defects. However, the fold of increase in the levels of granulins C and F is much higher than granulins A and B (Fig. 5b, Table S2), further supporting differential regulation of individual granulin peptides. In addition, we have shown that granulin A can be detected in the lysosome in both neurons and microglia and the levels of granulin A in microglia are regulated differently in response to different stimuli. In summary, the generation of antibodies specific to individual granulin peptides has yielded novel insights into the function of regulation granulins in the lysosome in physiological and pathological conditions.

## Material and methods

### Primary antibodies and reagents

The following antibodies were used in this study: mouse anti-GAPDH (Proteintech Group, 60,004–1-Ig), mouse anti-GFP (Proteintech Group), rat anti-mouse LAMP1 (BD Biosciences, 553,792), sheep anti-mouse PGRN (R&D Systems, AF2557) and rabbit anti IBA-1 (Wako, 01919741). Fluorescently labelled second antibodies were obtained from Li-Cor and Invitrogen.

To generate polyclonal antibodies to granulin peptides, granulin peptides were cloned in the pGEX6P-1 vector using restriction enzymes BamHI and PmeI (Table S1). The expression construct was transformed into the Origami bacterial strain (Novagen) and the expression of recombinant protein was induced with IPTG. The cells were lysed and lysates were incubated with GST beads. Bound proteins were eluted with glutathione. The buffer was exchanged to PBS using the Centricon devices (Millipore). Recombinant proteins were used to immunize rabbits using services provided by Pocono Rabbit Farm and Laboratory (Canadensis, PA).

The following reagents were also used in the study: Dulbecco’s modified Eagle’s media (DMEM) (Cellgro, 10–017-CV), 0.25% Trypsin (Corning, 25–053-CI), Papain (Worthington, LS003120), DNase I (Sigma D5025), Odyssey blocking buffer (LI-COR Biosciences, 927–40,000), Poly-D, L-ornithine (Sigma P0421), protease inhibitor (Roche, 05056489001), Pierce BCA Protein Assay Kit (Thermo scientific, 23,225) and O.C.T compound (Electron Microscopy Sciences, 62,550–01).

### Cell culture

HEK293T were maintained in Dulbecco’s Modified Eagle’s medium (Cellgro) supplemented with 10% fetal bovine serum (Gibco) and 1% Penicillin–Streptomycin (Invitrogen) in a humidified incubator at 37 °C and 5% CO_2_. Cells were transiently transfected with GFP tagged granulins using polyethylenimine as described [21]. Cells were harvested 2 days after transfection using ice cold RIPA buffer (150 mM NaCl, 50 mM Tris-HCl (pH 8.0), 1% Triton X-100, 0.5% sodium deoxycholate, 0.1% SDS) with 1 mM PMSF, proteinase and phosphatase inhibitors.

WT and *Grn*−/− mouse primary cortical neurons and microglia were isolated from P0 to P2 pups according to published protocols [16, 37].

### Mouse strains

C57/BL6 and *Grn*^−/−^ mice [38] were obtained from The Jackson Laboratory. *Ctsd−/−* [39], *Ctsb−/−* [40], *Ctsl−/−* [41], *Ctsb−/− Ctsl−/−* [33], *Ctsk−/−* [42] and *Ctsz−/−* [43] mice were characterized previously. PSAP knockout mice were previously described [44]. All animals (1–6 adult mice per cage) were housed in a 12 h light/dark cycle.

### Stroke and demyelination induction

Rose Bengal was used to induce stroke using a modified protocol [45]. Briefly, 4 months old mice were injected retro-orbitally with Rose Bengal (50 μl, 10 mg/ml) followed by 5 min illumination of the intact skull with a 561 nm laser. The skin was then sutured and mice were treated with 0.1 mg/kg Buprenorphine every 8 h for 24 h after surgery. Mice were allowed to recover for 7 days before perfusion and tissue collection.

Demyelination was induced by supplementing the diet with 0.2% (w/w) cuprizone (bis [cyclohexanone] oxaldihydrazone) in powdered rodent chow [46]. 10-week-old mice were fed a cuprizone supplemented diet for 5 weeks, during which the rodent chow (5 g/mouse/day) was replaced every other day. Untreated control mice were fed normal crushed chow for 5 weeks.

### Tissue preparation and western blot analysis

Mice were perfused with 1× PBS and tissues were dissected and snap-frozen with liquid nitrogen and kept at − 80 °C. On the day of the experiment, frozen tissues were thawed and homogenized on ice with bead homogenizer (Moni International) in ice-cold RIPA buffer (150 mM NaCl, 50 mM Tris-HCl (pH 8.0), 1% Triton X-100, 0.5% sodium deoxycholate, 0.1% SDS) with 1 mM PMSF, proteinase and phosphatase inhibitors. After centrifugation at 14,000×g for 15 min at 4 °C, supernatants were collected. Protein concentrations were determined via BCA assay, then standardized. Equal amounts of protein were mixed with loading buffer with fresh b-mercaptoethanol. Samples were separated by 4–12% Bis-Tris PAGE (Invitrogen) and transferred to 0.2 μm nitrocellulose. Western blot analysis was performed as described [15]. To quantify the levels of full-length PGRN or each granulin peptide (GRN), Image J software was used to measure the density of protein bands (75 kDa or 10 kDa). These values were normalized to GAPDH. To compare the levels of granulin in different tissues, the levels of granulins were first normalized to GPADH and then normalized to the value of liver, lung or spinal cord samples in the same gel.

### De-glycosylation assay

Spleen and/or liver lysates from WT mice were immunoprecipitated using anti-granulin B, C or E antibodies and the immunoprecipitates were treated with PNGase F (New England Biolabs) according to the manufacturer’s instructions.

### Immunofluorescence staining, image acquisition, and analysis

Mice were perfused with PBS and then 4% paraformaldehyde (PFA). The brains were removed and continuously postfixed overnight in 4% PFA at 4 °C. After being dehydrated, coronal brain sections (20 μm) were cut using a cryostat (Leica, Heidelberg, Germany) and processed for immunostaining. For granulin A staining, brain sections from *Grn*−/− mice were first blocked and permeabilized with 0.1% saponin in Odyssey blocking buffer followed by incubation with our homemade rabbit anti-mouse GRN-A antibody overnight at 4 °C. The supernatants were then collected to be used as primary antibody solution in subsequent immunostaining using brain sections. Tissue sections were blocked and permeabilized with 0.1% saponin in Odyssey blocking buffer before incubating with primary antibodies overnight at 4 °C. The next day, sections were washed with PBS three times followed by incubation with secondary fluorescent antibodies and Hoechst at room temperature for 2 h. The slides were then mounted using a mounting medium (Vector laboratories). Images were acquired on a CSU-X spinning disc confocal microscope (Intelligent Imaging Innovations) with an HQ2 CCD camera (Photometrics) using 100x objectives, 10 to 15 different random images were captured from the cortex or the corpus callosum region.

For the quantitative analysis of PGRN and GRN-A levels in microglia, the IBA1+ microglia were selected using the region of interest (ROI) tool after the data channels were separated (Image\Color\Split Channels). Next, PGRN or GRN-A signals within the IBA1 ROI were selected (Analyze\tools\ROI manager) and measured.

### Statistical analysis

All statistical analyses were performed using GraphPad Prism 8. All data are presented as mean ± SEM. Statistical significance was assessed by unpaired two-tailed Student’s *t*-test (for two groups comparison) or one-way ANOVA tests with Bonferroni’s multiple comparisons (for multiple comparisons). *P* values less than or equal to 0.05 were considered statistically significant. *, *p* < 0.05, **, *p* < 0.01, ***, *p* < 0.001, ****, *p* < 0.0001.

## Supplementary Information

**Additional file 1: Figure S1.** HEK293T lysates and media containing GFP or GFP tagged mouse granulins were probed with sheep anti-mouse PGRN antibodies from R&D systems to detect each individual granulin peptide. **Figure S2.** Quantification of the levels of individual granulins and the ratio between granulins and PGRN in the spleen, lung and kidney lysates from 4 to 5 months old WT and *Grn*^*−/−*^ mice. The value was normalized to that of liver sample on the same blot (set as 1). Data presented as mean ± SEM. *n* = 3. *, *p* < 0.05, **, *p* < 0.01, ***, *p* < 0.001, ****, *p* < 0.0001, unpaired two-tailed Student’s *t*-test. **Figure S3.** Analysis of PGRN and granulin levels in brain regions and spinal cord of female mice. (a) Western blot analysis of cortical and spinal cord lysates from 4 to 5 month old WT and *Grn*^*−/−*^ mice with antibodies against full length of PGRN and individual granulin A and C as indicated. (b) Western blot analysis of tissue lysates from 4.5 to 5 months old female WT mice with antibodies against full length of PGRN and individual granulin A and C as indicated. CX = cortex, Hp = hippocampus, CC = corpus callosum, Tha = thalamus, Cb = cerebellum, BS = brain stem, SC = spinal cord. (c) Quantification of experiment in (a). The ratio between total granulins to full length PGRN was quantified and normalized to that of spinal cord. *n* = 3–4. Data presented as mean ± SEM. **Table S1.** Sequences of mouse granulin peptides used in our study. **Table S2.** Summary in changes in PGRN levels and GRN/PGRN ratios in mice deficient in cathepsin or PSAP. n.s.c: No significant changes.

## Data Availability

The data supporting the findings of this study are included in the supplemental material. Additional data are available from the corresponding author on request. No data are deposited in databases.

## Abbreviations

FTLD: frontotemporal lobar degeneration
NCL: neuronal ceroid lipofuscinosis
PGRN: progranulin
PSAP: prosaposin
Cts: cathepsin
