# Supplementary material for: Differential regulation of progranulin derived granulin peptides
Source: Mol Neurodegener. 2022 Feb 4;17:15. doi: 10.1186/s13024-021-00513-9 (PMC8815130; doi:10.1186/s13024-021-00513-9)
Supplement: Supplementary file 1 — Additional file 1: Figure S1. HEK293T lysates and media containing GFP or GFP tagged mouse granulins were probed with sheep anti-mouse PGRN antibodies from R&D systems to detect each individual granulin peptide. Figure S2. Quantification of the levels of individual granulins and the ratio between granulins and PGRN in the spleen, lung and kidney lysates from 4 to 5 months old WT and Grn−/− mice. The value was normalized to that of liver sample on the same blot (set as 1). Data presented as mean ± SEM. n = 3. *, p < 0.05, **, p < 0.01, ***, p < 0.001, ****, p < 0.0001, unpaired two-tailed Student’s t-test. Figure S3. Analysis of PGRN and granulin levels in brain regions and spinal cord of female mice. (a) Western blot analysis of cortical and spinal cord lysates from 4 to 5 month old WT and Grn−/− mice with antibodies against full length of PGRN and individual granulin A and C as indicated. (b) Western blot analysis of tissue lysates from 4.5 to 5 months old female WT mice with antibodies against full length of PGRN and individual granulin A and C as indicated. CX = cortex, Hp = hippocampus, CC = corpus callosum, Tha = thalamus, Cb = cerebellum, BS = brain stem, SC = spinal cord. (c) Quantification of experiment in (a). The ratio between total granulins to full length PGRN was quantified and normalized to that of spinal cord. n = 3–4. Data presented as mean ± SEM. Table S1. Sequences of mouse granulin peptides used in our study. Table S2. Summary in changes in PGRN levels and GRN/PGRN ratios in mice deficient in cathepsin or PSAP. n.s.c: No significant changes. [file 13024_2021_513_MOESM1_ESM.docx]

**Supplemental Material**

**
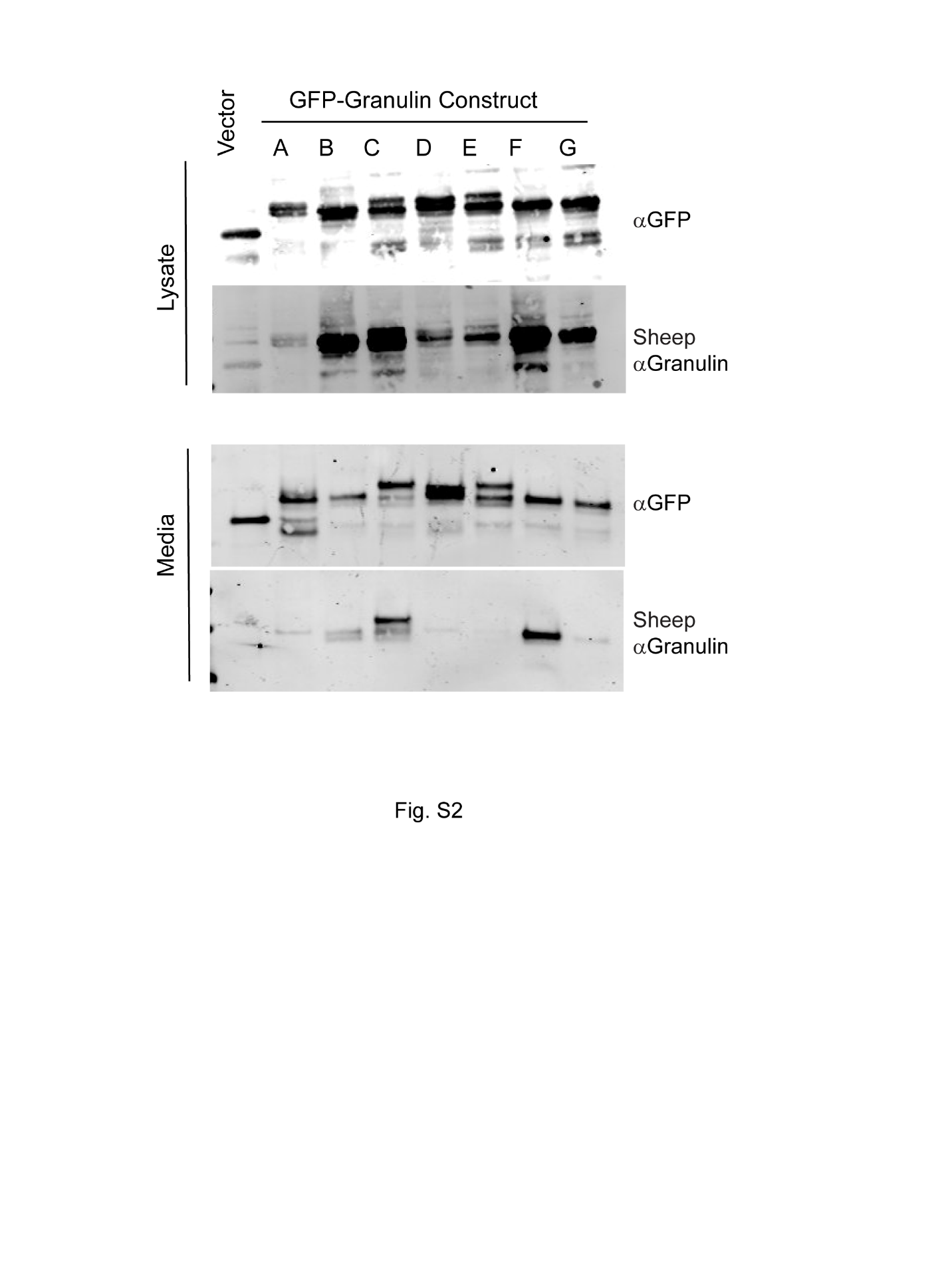
**

**Figure S1:** HEK293T lysates and media containing GFP or GFP tagged mouse granulins were probed with sheep anti-mouse PGRN antibodies from R&D systems to detect each individual granulin peptide.

**
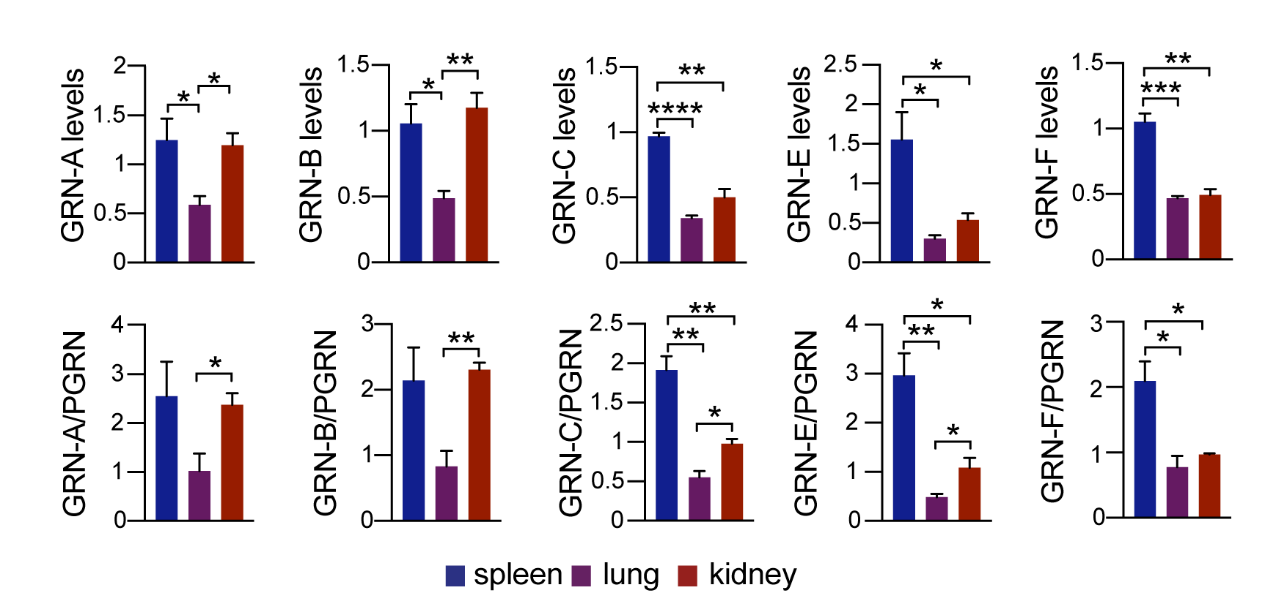
**

**Figure S2:** Quantification of the levels of individual granulins and the ratio between granulins and PGRN in the spleen, lung and kidney lysates from 4-5 months old WT and *Grn^-/-^* mice. The value was normalized to that of liver sample on the same blot (set as 1). Data presented as mean ± SEM. n = 3. *, p<0.05, **, p<0.01, ***, p<0.001, ****, p<0.0001, unpaired two-tailed Student’s *t*-test.


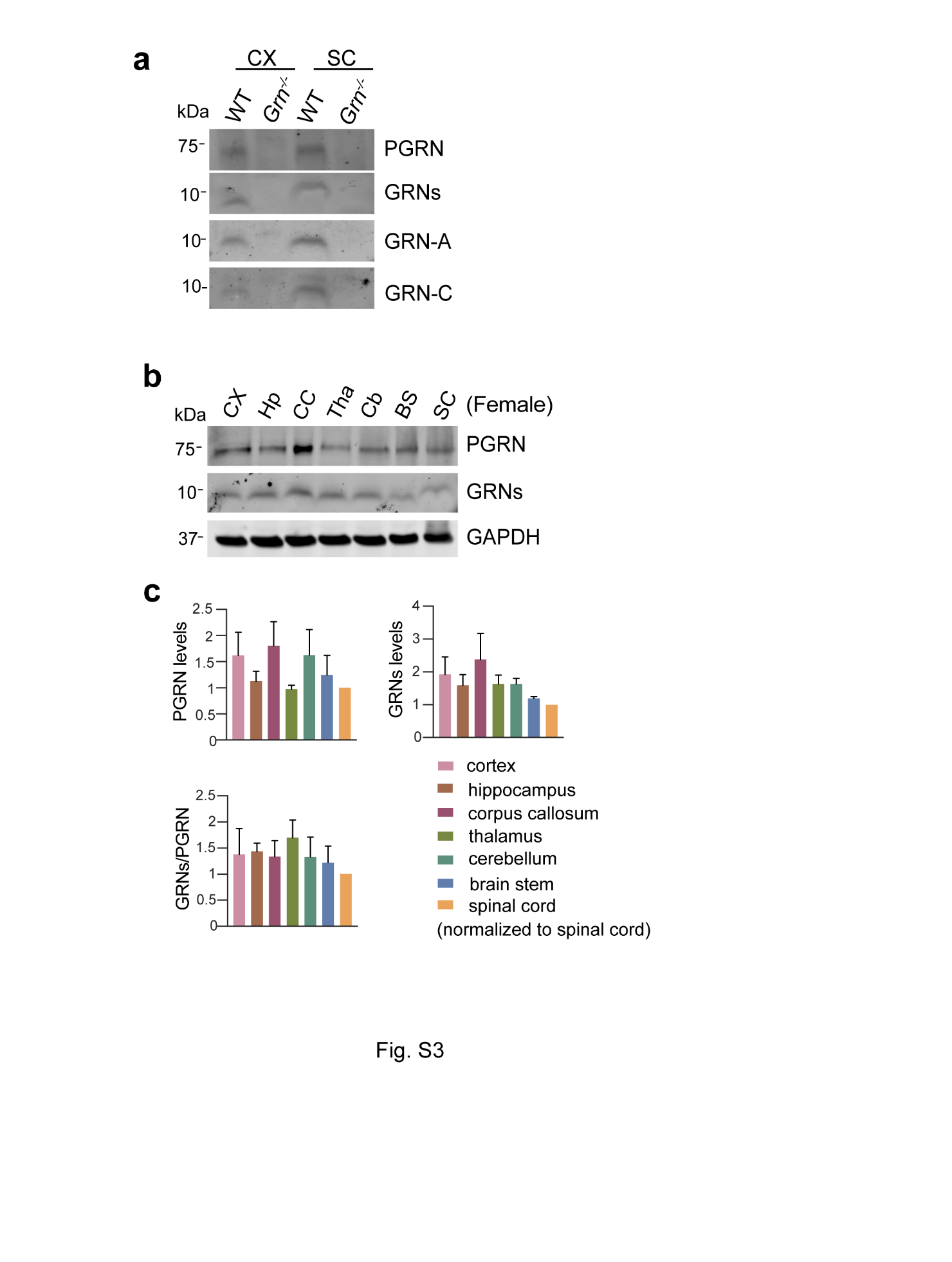


**Figure S3: Analysis of PGRN and granulin levels in brain regions and spinal cord of female mice.**

##### Western blot analysis of cortical and spinal cord lysates from 4-5 month old WT and *Grn^-/-^* mice with antibodies against full length of PGRN and individual granulin A and C as indicated.

(b) Western blot analysis of tissue lysates from 4.5 to 5 months old female WT mice with antibodies against full length of PGRN and individual granulin A and C as indicated. CX = cortex, Hp = hippocampus, CC = corpus callosum, Tha = thalamus, Cb = cerebellum, BS = brain stem, SC = spinal cord.

(c) Quantification of experiment in (a). The ratio between total granulins to full length PGRN was quantified and normalized to that of spinal cord. n = 3-4. Data presented as mean ± SEM.

**Table S1: Sequence of mouse granulin peptides used in our study.**

| **GRN** | **Sequence** |
| --- | --- |
| A | VKCDMEVSCPEGYTCCRLNTGAWGCCPFAKAVCCEDHIHCCPAGFQCHTEKGTCEM |
| B | VVCPDAKTQCPDDSTCCELPTGKYGCCPMPNAICCSDHLHCCPQDTVCDLIQSKCLS |
| C | TPCDDFTRCPTNNTCCKLNSGDWGCCPIPEAVCCSDNQHCCPQGFTCLAQGYCQK |
| D | IGCDQHTSCPVGQTCCPSLKGSWACCQLPHAVCCEDRQHCCPAGYTCNVKARTCEK |
| E | VECGEGHFCHDNQTCCKDSAGVWACCPYLKGVCCRDGRHCCPGGFHCSARGTKCLR |
| F | VQCPGSQFECPDSATCCIMVDGSWGCCPMPQASCCEDRVHCCPHGASCDLVHTRCVS |
| G | GSCQTHGHCPAGYSCLLTVSGTSSCCPFSKGVSCGDGYHCCPQGFHCSADGKSCFQ |

**Table S2: Summary in changes in PGRN levels and GRN/PGRN ratios in mice deficient in cathepsin or PSAP.**

| Genotypes | PGRN  levels | GRN-A  /PGRN | GRN-B  /PGRN | GRN-C  /PGRN | GRN-F  /PGRN |
| --- | --- | --- | --- | --- | --- |
| *Ctsb^-/-^* | n.s.c |  |  | n.s.c | n.s.c |
| *Ctsk^-/-^* | n.s.c | n.s.c | n.s.c | n.s.c | n.s.c |
| *Ctsz^-/-^* | n.s.c | n.s.c | n.s.c | n.s.c | n.s.c |
| *Ctsd^-/-^* |  |  |  |  | n.s.c |
| *Ctsl^-/-^* | n.s.c | n.s.c | n.s.c | n.s.c | n.s.c |
| *Ctsb^-/-^Ctsl^-/-^* |  | n.s.c | n.s.c | n.s.c | n.s.c |
| *Psap^-/-^* |  |  |  |  | n.s.c |

n.s.c: No significant changes.
